# Differential Impact of Temperature, Release Rate, Prey Density, and Pesticides on *Hyperaspis trifurcata* (Coleoptera: Coccinellidae) to Optimize Integrated Management of *Dactylopius opuntiae* (Hemiptera: Dactylopiidae)

**DOI:** 10.3390/plants14071129

**Published:** 2025-04-05

**Authors:** Rachid Bouharroud, Salahddine Chafiki, Redouan Qessaoui, Yassine Imlil, Jamila Bargach, Aissa Derhem, Rachid Elaini

**Affiliations:** 1 Regional Center of Agricultural Research of Agadir, National Institute of Agricultural Research, Avenue Ennasr, BP415 Rabat Principale, Rabat 10090, Morocco; salahddine.chafiki@um6p.ma (S.C.); redouan.qessaoui@inra.ma (R.Q.);; 2 AgroBioSciences Department (AgBS), Mohammed VI Polytechnic University (UM6P), Ben Guerir 43150, Morocco; 3 Dar Si Hmad Foundation, Agadir 80000, Morocco; j.bargach@darsihmad.org (J.B.); a.derhem@darsihmad.org (A.D.); 4 Department of Mathematics and Computer Science, Mohammed I University, Oujda 60000, Morocco; 5 Integrated Pest Management Department, Omnium Agricole du Souss, Agadir 80000, Morocco; elaini@groupesaoas.com

**Keywords:** classical biological control, *Hyperaspis trifurcata*, *Dactylopius opuntiae*, survival test, pesticide side effects, NENA region

## Abstract

The current work aims to establish an integrated pest management strategy using *Hyperaspis trifurcata* Schaeffer (Coleoptera: Coccinellidae) to control *Dactylopius opuntiae* Cockerell (Hemiptera: Dactylopiidae) and to assess the side effects of pesticides commonly used on this predator. The first part of this study was performed under controlled conditions at two temperatures with three prey densities and two release rates for 83 days. Under field conditions, a survival time test was conducted in a screen house (1.2 ha), where a total of 5700 predators were released on 1425 cactus plants and then monitored for a period of 23 weeks. Furthermore, eight pesticides were tested on *H. trifurcata* in laboratory conditions at five rates in order to define the lethal doses. Under controlled conditions, the effect of temperature on predation was not significant until 27 days after release. However, the prey density significantly impacted the predation rates from the 10th to 27th day after release (*p* < 0.001). The predator release rate significantly affected predation starting from the 15th day after release. The lowest median survival time based on Kaplan–Meier tests was obtained at 30 °C (the high temperature) for eight predators/cladode (27 days), but the highest was at 26 °C (the low temperature) for four predators/cladode (63 days). Depending on cochineal infestation, the effect of temperature significantly increased the predation rate from the 10th to 49th day after release, but only at a high density (50 colonies/cladode). Under field conditions, the effect of the infestation level on the survival function was significant (Log-Rank *p* < 0.05), and the median times were 111 and 130 days after release for low and high densities, respectively. Acetamiprid, Vaseline oil, black soap, copper oxychloride, and paraffin oil were highly toxic to *H. trifurcata* (>84% of mortality), and the LD_50_ values ranged from 2.3 to 69.6% of the recommended rate. For Mancozeb at the recommended dose, the mortality rate was low (<2%). The large-scale release of *H. trifurcata* would be successful in the Near East and North Africa (NENA) region, provided that the use of the mentioned pesticides is avoided or at least reduced.

## 1. Introduction

Cactus pear, belonging to the *Opuntia* spp. genus, is not native to the Mediterranean region, where it was introduced within the last three centuries [1]. *Dactylopius opuntiae* (Hemiptera: Dactylopiidae), or wild cochineal scale, is considered a primary pest of *Opuntia ficus-indica* (L.) Miller (Caryophyllales: Cactaceae) in many countries [2]. In 2014, this scale insect was detected for the first time in Morocco, where extensive damage was caused in several cactus-producing areas in the country [3]. Chemical control has been the main method adopted to manage this pest. However, this approach leads to harmful effects on human and animal health and the environment [4,5], in addition to the emergence of resistance [6]. Therefore, many alternative management strategies have been explored, such as the use of resistant genotypes, detergents, bioactive compounds, and biological control agents [7,8,9]. However, today, the most sustainable method would be an integrated pest management program based on classical biological control using predators since no parasitoids have been reported for this cochineal at its center of origin. Several species of predators, mainly Coccinellidae *Chilocorus cacti* Linnaeus (Coleoptera: Coccinellidae), *Hyperaspis trifurcata* (Coleoptera: Coccinellidae), and *Cryptolaemus montrouzieri* Mulsant (Coleoptera: Coccinellidae), have been reported as the most effective [10,11,12,13]. *Hyperaspis trifurcata* has been shown to be a native predator of the wild cochineal scale. It has been reported that *H. trifurcata* females can consume more than 5000 *D. opuntiae* nymphs during their lifetime and prey on this cochineal at all developmental stages [9]. In Morocco, 13 predators were reported to be able to affect the cactus cochineal [8,14]. However, their frequency, specificity, and voracity were very low compared to those of the three most interesting predators, *Leucopina bellula* Williston (Diptera: Chamaemyiidae), *H. trifurcata*, and *Laetilia coccidivora* Comstock (Lepidoptera: Pyralidae), as reported in several studies [15,16,17]. In fact, among the three predators, *H. trifurcata* was the most effective one to be used as a biological control agent in the Mediterranean area where *D. opuntiae* was reported; this was attributed to its longer life cycle, which ranges between 64 and 75 days, with more than 39 days as an adult [18], and the low concentration of carminic acid in the body of female *D. opuntiae* (2 to 5%) compared to *Dactylopius coccus* Costa (Hemiptera: Dactylopiidae) (8 to 25%), as reported by Barreto-García et al. [19] and Aldama-Aguilera et al. [20].

The main objectives of this study are to evaluate the effectiveness of *H. trifurcata* in controlling *D. opuntiae* under field conditions and to optimize its integration with other control methods, such as chemical control, for a holistic approach.

## 2. Results

### 2.1. Effects of Temperature, Prey Density, and Predator Release Rate Under Controlled Conditions

Regardless of the cochineal density, the speed of *D. opuntiae* control varied over the period from predator introduction to the end of the experiment. In fact, the 27th day after *H. trifurcata* introduction was a key time point for the biological control of *D. opuntiae*, as this was when a significant impact was first observed under conditions of 30 °C and a predator release rate of eight per cladode. Then, 35 days after *H. trifurcata* introduction was needed to completely destroy *D. opuntiae* cochineals at 50 colonies per cladode. No significant effect was observed six days after the release of *H. trifurcata* on infested cladodes. Ten days after release, the first impact of *H. trifurcata* predation was observed (Table 1) at prey densities (*p* < 0.001) of 10 and 50 cochineals per cladode. Among the three parameters studied (temperature, prey density, and predator release rate), the temperature had no significant effect (*p* > 0.05) on the mean number of surviving cochineals during the first 3 weeks after release. Twenty-seven days after release, the effects of the three studied parameters were significant (*p* < 0.05). In fact, during the same period after release, an average of 47.00 surviving cochineals was observed for a predator release rate of eight per cladode at a temperature of 26 °C. However, only 7.00 survivors were observed at 30 °C for cladodes containing 50 cochineals before release. In addition, at four predators per cladode, the temperature had a significant visual effect for the same cochineal density (50/cladode), and an average of only 8.67 cochineals were alive after 27 days at 30 °C, compared to 48.5 at 26 °C. The obtained results show that the predator release rate at 26 °C did not affect the three prey densities (10, 20, and 50 cochineals/cladode). However, at 30 °C, a difference between the two predator release rates was clearly observed at the three prey densities. In fact, from 35 to 49 days after *H. trifurcata* release, the prey density had no significant effect (*p* > 0.05) on the number of survivors, in contrast to the temperature (*p* < 0.01) and predator release rate (*p* < 0.01). The most important difference was observed at 50 cochineals per cladode, where a mean of 31 survivors was observed for four *H. trifurcata* per cladode at 26 °C, compared to 3.67 for eight predators per cladode and 1 to 0, respectively, at 30 °C. The three parameters studied were no longer significant 63 to 83 days after *H. trifurcata* release since the mean number of cochineal survivors in all experimental units ranged from 0 to 1.67 per cladode regardless of the initial prey density (cochineals/cladode). The four probable interactions (Temperature × Prey density, Temperature × Predator release rate, Prey density × Predator release rate, and Temperature × Prey density × Predator release rate) between the three studied parameters were significant from the 10th to 49th day after release (*p* < 0.05); even the temperature effects only became significant 27 days after *H. trifurcata* release (Table 1).

**Figure 1:**
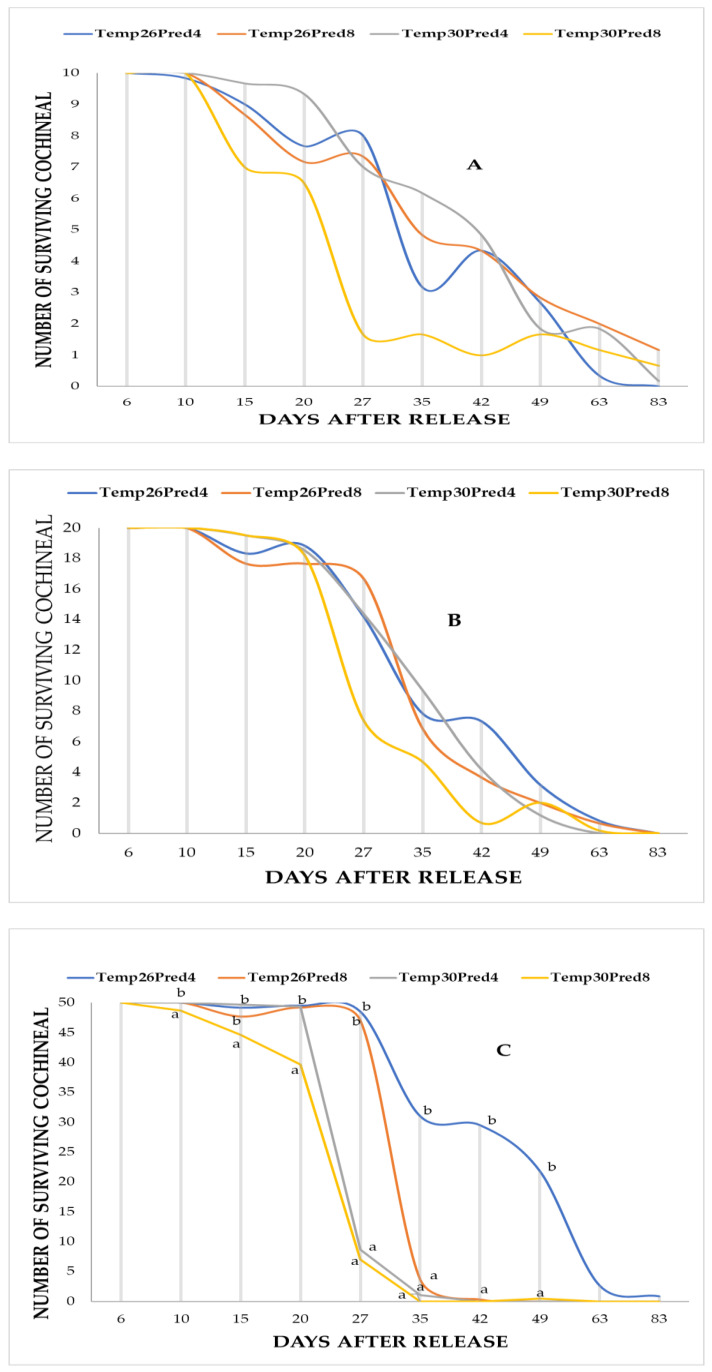
Mean number of surviving *D. opuntiae* at 3 prey densities over 83 days after release of *H. trifurcata* (4 and 8 predators/cladode) and at 2 temperatures (26 and 30 °C). (**A**): 10 colonies/cladode; (**B**): 20 colonies/cladode; and (**C**): 50 colonies/cladode. Means with the same letter are not significantly different at *p* ≤ 5% according to the Newman-Keuls test.

**Figure 2:**
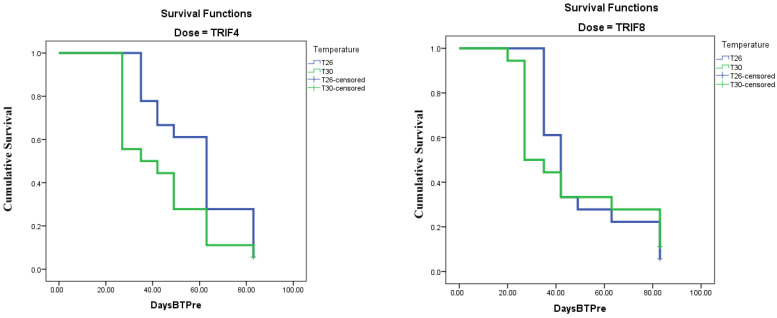
Survival function curves for 2 tested release rates (TRIF4 and TRIF8) at 2 constant temperatures (26 and 30 °C) varying with number of days before total predation (DaysBTPre).

The Kaplan–Meier survival analysis under controlled conditions in separate rooms at both temperatures (26 and 30 °C) showed the significant effects of temperature and the release rate (*p* = 0.007 and 0.026 for Breslow and Tarone–Ware tests, respectively) on the predation effect of *H. trifurcata* at three prey densities of *D. opuntiae*. Indeed, the shortest (27 days) median time to total predation was obtained at a temperature of 30 °C with eight *H. trifurcata* individuals released, and the longest median duration to total predation was 63 days at a temperature of 26 °C with four predators released. At 30 °C with four predators per cladode and at 26 °C with eight predators, the median survival times observed were 35 and 42 days, respectively (Table 2 and Table 3, Figure 1 and Figure 2).

### 2.2. Pesticide Side Effects on H. trifurcata

The probit analysis showed that the lowest LD_50_ was obtained using Acetamiprid (2.3% of RR), followed by Vaseline oil and Potassium salt of fatty acids, with 6.3 and 6.8%, respectively. The less toxic pesticide was Pyriproxyfen, with an LD_50_ of 294% of the recommended rate. The LD_50_ for Mancozeb was not obtained in the probit analysis because the mortality rates indicated that it was not toxic, even at 100% of the recommended rate (Table 4 and Table 5). The mortality rates (*p* < 0.001; F = 54.505) caused by Acetamiprid, Potassium salt of fatty acids, paraffin oil, copper oxychloride, and Vaseline oil at their recommended rates ranged from 84 to 100%.

### 2.3. Field Efficacy of H. trifurcata on D. opuntiae

**Figure 3:**
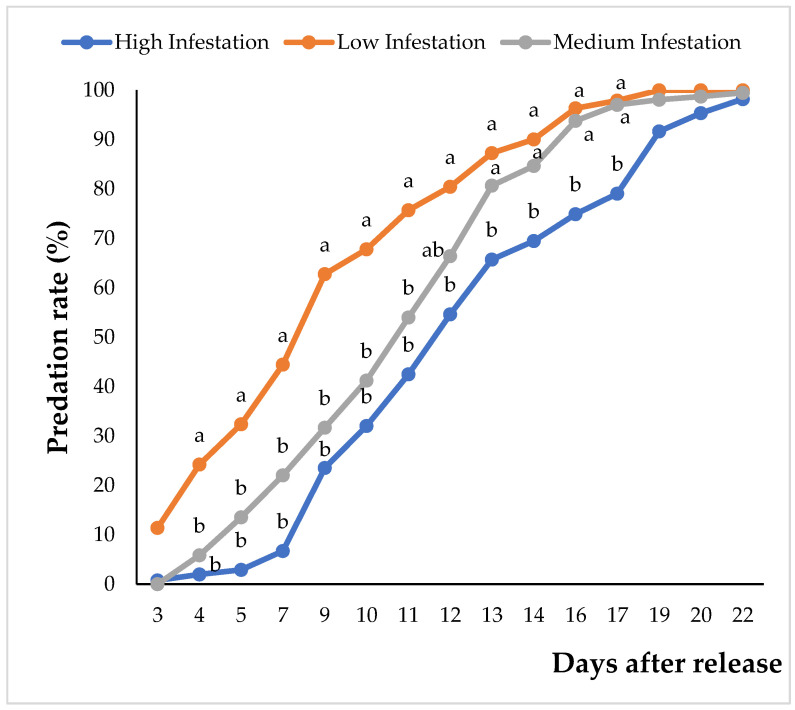
Predation rate evolution using *H. trifurcata* to control *D. opuntiae* for 22 weeks under field conditions. Mean predation rates with the same letters on the same monitoring days after release were not significantly different based on N-K means comparison at *p* < 5%.

**Figure 4:**
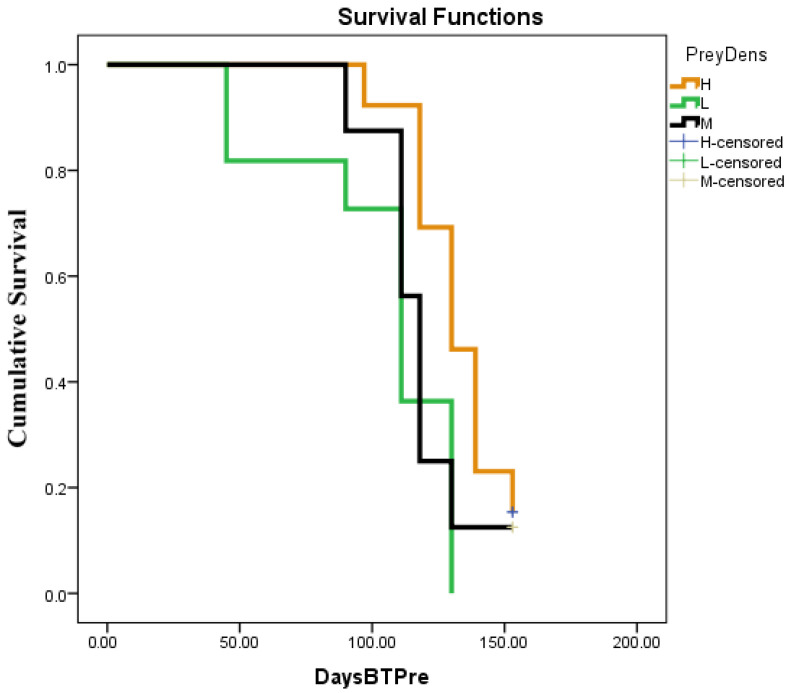
Survival function curves for *H. trifurcata* predation under field conditions depending on cochineal density per cladode with number of days before total predation (DaysBTPre). H: high (more than 100); M: medium (from 51 to 100); and L: low (less or equal to 50).

The first predation impact was observed 19 days (3rd week) after the release of *H. trifurcata*, but it was not statistically significant (*p* > 0.05). In fact, the first significant effect (*p* < 0.01) was observed during the fourth week, and the most important predation rate was observed on cladodes with low cochineal infestation, even though this rate was quite low at 24%. From the 5th to 11th week, the predation rate increased gradually with the same trend for three infestation levels, forming two different groups: the first with high and medium infestation and the second with low infestation (Figure 3). The predation rates ranged from 3 to 42%, 14 to 54%, and 32 to 76% for high, medium, and low infestation, respectively. During the 12th week, the results of medium infestation seemed to be approaching those of the low-infestation group, forming three separate groups. The predation rates of *H. trifurcata* on *D. opuntiae* were 55, 66, and 80% for high, medium, and low infestation, respectively. From the 13th to the 17th week after release, the predation rates continued to increase significantly. However, during this period, the low and medium infestations were considered to be statistically within the same group, and predation rates ranged from 87 to 98% and 81 to 97%, respectively. The predation rate in the high-infestation group ranged from 66 to 79%. After the 17th week, the predation rates exceeded 91%, although there was no significance (*p* > 0.05) of the effect of cochineal density on the predation rates. As shown in Table 6 and Table 7 and Figure 4, the effect of infestation levels on the survival function is significant (Log-Rank *p* < 0.05), and the medians of the survival curves were 111, 118, and 130 days after *H. trifurcata* release for low, medium, and high cochineal infestation levels, respectively (Table 6 and Table 7, Figure 4).

## 3. Discussion

The results confirm that the prey density is the first significant factor affecting the predation efficiency of *H. trifurcata* on *D. opuntiae*, especially at the beginning of the trial (10 days after release). Then, 2 weeks after release, the predator release rate impacted the predation rate. The effect of temperature was not observed until the 27th day following release. The temperature mainly affects reproductive and development rates, that is, copulation and offspring (females’ fecundity and fertility). The impacts of the prey density and predator release rate were faster than that of temperature. Ascencio Contreras [21] showed that natural enemies associated with *D. opuntiae*, including *H. trifurcata*, were positively correlated with the *D. opuntiae* population. A mixed diet composed of all stages of *D. opuntiae* seems to be beneficial for the development of *H. trifurcata*. Vanegas-Rico et al. [9] reported that *H. trifurcata* fertility and fecundity were reduced when using only the first two larva stages of *D. opuntiae* as a diet. The voracity of *H. trifurcata* on *D. opuntiae* is important since this coccinellid consumes more than 5000 nymphs. Vanegas-Rico et al. [9] showed that the maximum predation rate of fourth-instar *H. trifurcata* on *D. opuntiae* nymphs was observed on the 21st day (69 nymphs) and on the 32nd day for newly emerged adults (152 nymphs).

Eisner et al. [22] reported that *H. trifurcata* has the ability to tolerate carminic acid, which is why *Dactylopius* spp. are considered suitable prey. The oviposition behavior is characterized by the tendency to lay eggs on wax as a chosen target site, with a particular preference for the wax of gravid females [9]. This phenomenon could explain our observation in the low-prey-density treatment (10 colonies/cladode), where the period needed for total predation was longer than in the high-prey-density group (50 colonies/cladode). Cruz-Rodríguez et al. [15] confirmed in a field study that the predation rate was positively correlated with *D. opuntiae* population abundance. It has been reported that cannibalism can occur in other coccinellids when prey is deficient [23,24,25]. The prey density impacted the fertility of *Chilocorus nigritus* Fabricius (Coleoptera: Coccinellidae) feeding on the scale insect *Abgrallaspis cyanophylli* Signoret (Hemiptera: Diaspididae), but this was transient [26]. Integrated pest management principles are based on an economic threshold, not total eradication, and for cacti, a balance should be expected, as reported in Mexico by Cruz-Rodríguez et al. [15]. The authors referred to this balanced ecosystem as “autonomous biological control”, where, over 10 years, no chemical control methods were adopted since the Mexican economic threshold related to *D. opuntiae* (30% of cladodes with 10 colonies) was not reached [27]. For the ladybird *Hyperaspis campestris* Herbst (Coleoptera: Coccinellidae), 1000 individuals per Ha were released to control the population of cottony camellia scale *Pulvinaria floccifera* Westwood (Hemiptera: Coccidae), and the expected efficiency below the threshold was observed within 2 to 3 years after release [28]. In Israel, individuals of *H. trifurcata* were observed 21 km away from the release site after 18 months [29]. This is of great importance for reaching areas that are difficult to access, such as cacti established in spontaneous plantations (e.g., mountains). Other species from the *Hyperaspis* genus seem to increase their development and reproductive parameters at high prey densities, as confirmed by Reyd and Le Rü [30] for *Hyperaspis raynevali* Mulsant (Coleoptera: Coccinellidae) on the cassava mealybug *Phenacoccus manihoti* Matile-Ferrero (Hemiptera: Pseudococcidae). According to Seyfollahi et al. [31], *Hyperaspis polita* Weise (Coleoptera: Coccinellidae) could successfully survive, develop, and reproduce on the cotton mealybug *Phenacoccus solenopsis* Tinsley (Hemiptera: Pseudococcidae) in a wide range of temperatures from 25 to 35 °C, with an optimal temperature of 30 °C. The Harlequin ladybird *Harmonia axyridis* Pallas (Coleoptera: coccinellidae) was used at 50 individuals/plant to control the hop aphid *Phorodon humuli* Schrank (Hemiptera: Aphididae) [32].

Crowder [33] discussed the effect of increasing release rates on the success of biological control and reported 12 cases in which the release rate had no effect, compared to 7 in which increasing the release rate had a positive effect on biological control. Indeed, under controlled conditions, the median survival times were lower than those obtained under field conditions (111 to 130 days), and the duration required for total predation was also low, ranging between 27 and 83 days, depending on the prey density. Predators in controlled conditions have no choice, and adults have to consume what is available, but in field conditions, the coccinellids are free to move on to other cladodes depending on the infestation level. The inverse prey density effect was observed in the field compared to controlled conditions. It seems that the availability of mixed stages of *D. opuntiae* at a high density compared to low and medium densities was a key factor in the predation rate, as reported by Vanegas-Rico et al. [9]. In fact, in the current study, *H. trifurcata* adults grouped or aggregated on one colony while leaving other colonies free (Bouharroud, personal communication) at a low prey density but not at a high density. As reported by Juliano [34], the prey density is negatively related to predator efficiency for the type II functional response of *H. trifurcata* and shows the ability of the predator to deal with a prey population rise.

The pesticides tested on *H. trifurcata* showed the possible failure of an IPM program when using the neonicotinoid insecticide Acetamiprid (LD_50_ = 2.3% of recommended rate), which is reported by many studies for its sublethal effects on natural enemies [35]. For *H. trifurcata* release, Vaseline oil and Potassium salt of fatty acids (LD_50_ = 6.3 and 6.85% of recommended rate, respectively) should be avoided to prevent a negative effect on *D. opuntiae* biocontrol. The action of Vaseline oil is made easier by its lipophilic properties and rapid penetration through insect cuticles [36], and Potassium salt of fatty acids also acts on the cuticle via hemolytic action, causing the breakdown of cell membranes [37]. Several questions remain unclear: Why is this behavior predicted? Is it dependent on the prey or the predator? If volatiles are involved, what are they? Is there a choice for oviposition colonies and predation colonies? When there is no prey on-site, what is the distance to cover or speed of movement in large-scale releases?

## 4. Materials and Methods

### 4.1. Effect of Temperature, Prey Density, and Predator Release Rate Under Controlled Conditions

Selected infected cladodes were potted on a substrate composed of sand and peat at 1 to 3 parts (*v*/*v*). The pots (5 L) were watered moderately and as needed in order to prevent cladode rot. Three prey densities were tested (10, 20, and 50 colonies of *D. opuntiae* per cladode). The cladodes were collected from an infested field cactus, *O. ficus-indica*, and transported the same day to the insectary. To estimate the appropriate needs of predators for future programs, two release rates (4 and 8 adults per cladode) of the predator *H. trifurcata* were adopted to test the predatory effect at 2 temperatures (26 and 30 °C). Indeed, 30 °C was chosen in order to check the efficiency of the predator since growth rates are more beneficial for *D. opuntiae* than for *H. trifurcata* at moderately high temperatures. Thus, the predation potential of *H. trifurcata* was evaluated when its unique prey (*D. opuntiae*) was more advantaged. The trial was conducted using controlled room parameters for relative humidity (65 ± 5%) and a photoperiod of 16/8 h for dark and light. The rooms (26 and 30 °C) were equipped with shelves containing 3 separate netted steel racks 120 × 60 × 180 cm (L/l/h). The dynamics of the predator and prey population were monitored for 83 days after *H. trifurcata* introduction until no surviving cochineals were observed. The split-plot experimental design was adopted to perform experiments with 3 factors: i. temperature (26 and 30 °C); ii. prey density (10, 20, and 50 colonies per cladode); and iii. predator release rate (4 and 8 adults per cladode) at a *p* < 5% level with 6 replicates.

### 4.2. Field Efficiency of H. trifurcata on D. opuntiae

This part of the study was conducted in a screen house of 1.2 ha. The plot comprises 22 rows divided into 3 blocks. Four adults of *H. trifurcata* per plant were released in the screen house of 1.2 ha. A total of 5700 predators were released with a sex ratio of 1 male to 1 female on 1425 cochineal-infested cactus plants (4 predators/plant). The infestation was natural, and an initial evaluation of the prey density was performed before *H. trifurcata* release, which showed a range of infestation levels, from low (less than or equal to 50) to medium (from 51 to 100) to high (more than 100 individuals of all mixed stages per cladode). A sample of 12 to 16 infested cladodes was selected in each block, which is equivalent to a total of 40 cladodes. Since infestation was natural, infested cladodes were randomly selected based on their infestation level. For each block, at least 4 cladodes represented each infestation level, and predation potential was checked throughout the period of the experiment (153 days). Each cladode was labeled, and pictures were taken weekly in order to prevent disturbing cochineals and *H. trifurcata* for 22 weeks (23 observations). The smartphones used have a resolution of 48 megapixels, and the pictures were taken from the same distance, with the same orientation and zoom, and at the same time of day (from 08:30 to 11:30 am). Before *H. trifurcata* release, the number of cochineal colonies on each cladode was counted, and then the number of survivors was monitored during the experiment. Climate data were monitored using an iMetos electronic weather station (Pessl Instruments, Weiz, AUSTRIA). Minimum, maximum, and mean temperatures (°C) and mean relative humidity (%) were recorded during the experiment. The adopted experimental design was a randomized complete block.

### 4.3. Pesticide Side Effects on H. trifurcata

Eight pesticides were tested at 5, 20, 50, 80%, and 100% of their recommended rates, as shown in Table 8. The concentrations were prepared using distillate water and polyoxethylene sorbitan monolaurate (Tween 20). The control was sprayed with distillate water and Tween 20.

Toxicity to *H. trifurcata*: Ten adults (3 ± 1 days) of *H. trifurcata* (sex ratio 1:1) were introduced into plastic (diameter = 85 mm) ventilated Petri dishes with a hole (diameter = 20 mm) in the lid covered by a glued piece of insect netting. The sprayed coccinellids were fed ad libitum with mixed waxy females and nymphs of *D. opuntiae* after one day of fasting. Six replicates were used for each concentration, and the bioassay was replicated twice. Then, for each concentration, 120 adults were tested, and for the whole experiment, the total number of tested adults was 10 × 6 × 6 × 2 × 8 (ladybird × concentration × replicate × bioassay × pesticide) = 5760 adults. The bioassay was conducted at laboratory ambient temperature. The mortality rates were monitored for 3 days after the spray.

### 4.4. Statistical Analysis

IBM SPSS software Version 23 was adopted to perform ANOVA and Newman–Keuls for mean comparisons at a level of *p* < 5%. The survival analysis of the field efficiency of *H. trifurcata* on *D. opuntiae* was performed using the Kaplan–Meier test at a critical level of significance for Log-Rank, Breslow, and Tarone–Ware (*p* < 5%). A probit analysis of the concentration-dependent mortality data was conducted using POLO-PC (LeOra Software, 1987, Berkeley, CA, USA). The following parameters were estimated: LD_10_, LD_50_, LD_90_, fiducial limits, standard error, and slope of regression.

## 5. Conclusions

The results clearly show that biological control using *H. trifurcata* to manage the wild prickly pear cochineal *D. opuntiae* will achieve a balance and protect cactus plantations in the NENA region. The current work contributes to the successful management of *D. opuntiae* by defining parameters that improve or impair classical biological control of *H. trifurcata*, such as temperature, prey density, release rate, pesticide side effects, and their interactions. In fact, the survival time is a key input for a decision support system.

## Figures and Tables

**Table 1:** Significance (*p*-values) of effects of temperature, prey density, and predator release rate and their interactions on the predation rate of *H. trifurcata* on *D. opuntiae*.

Days after the introduction of *H. trifurcata*	6	10	15	20	27	35	42	49	63	83
Temperature	-	0.072	0.848	0.099	0.000	0.004	0.000	0.005	0.208	0.466
Prey density	-	0.000	0.000	0.000	0.000	0.109	0.144	0.090	0.216	0.309
Predator release rate	-	0.072	0.000	0.005	0.022	0.002	0.000	0.014	0.527	0.602
Temperature × Prey density	-	0.010	0.033	0.021	0.000	0.001	0.004	0.019	0.298	0.812
Temperature × Predator release rate	-	0.022	0.051	0.037	0.018	0.156	0.017	0.016	0.800	0.917
Prey density × Predator release rate	-	0.010	0.027	0.106	0.858	0.016	0.008	0.009	0.216	0.155
Temperature × Prey density × Predator release rate	-	0.025	0.135	0.050	0.660	0.001	0.000	0.004	0.072	0.515

**Table 2:** Mean and median survival times for total predation under controlled conditions.

Release Rate (Ind./Cladode)	Temperature (°C)	Mean (Days)	Std. Err	95% Confidence Interval		Median (Days)	Std. Err	95% Confidence Interval	
4	26	59.22	4.42	50.56	67.89	63.00	4.43	54.31	71.69
	30	44.17	4.65	35.05	53.28	35.00	15.91	3.82	66.18
8	26	49.94	4.59	40.95	58.94	42.00	2.80	36.51	47.49
	30	46.28	5.98	34.56	57.99	27.00	3.54	20.07	33.93

**Table 3:** Test of equality of survival distributions at different temperatures.

	Chi-Square	df	Significance
Log-Rank (Mantel–Cox)	2.576	1	0.109
Breslow (Generalized Wilcoxon)	7.348	1	0.007
Tarone–Ware	4.962	1	0.026

**Table 4:** Mortality rates caused by 8 pesticides at their recommended rates on *H. trifurcata* adults 48 h after treatment.

Active Ingredient	Mean Mortality Rate (%) *	Std. Error	95% Confidence Interval	
			Lower Bound	Upper Bound
Acetamiprid	100.00 ^d^	4.82	90.23	109.78
Copper oxychloride	90.00 ^d^	4.82	80.23	99.78
Mancozeb	1.67 ^a^	4.82	−8.11	11.44
Paraffin oil	90.00 ^d^	4.82	80.23	99.78
Pyriproxyfen	28.00 ^b^	5.29	17.29	38.71
Potassium salt of fatty acids	100.00 ^d^	5.29	89.29	110.71
Vaseline oil	84.00 ^d^	5.29	73.29	94.71
White oil	58.33 ^c^	4.82	48.56	68.11

* Mortality rates followed by the same letters are not significantly different at *p* ≤ 5%.

**Table 5:** Probit analysis of log-mortality responses 48 h after treatment.

Pesticides	Lethal Doses (%RR *)		Confidence Limits	Slope	Standard Error	Log (L)	Heterogeneity	g	Number of Individuals	#Controls
White oil	LD_10_	6.66	1.645 to 12.768	1.406	0.234	179.3	1.31	0.153	298	60
	LD_50_	54.33	37.735 to 79.125							
	LD_90_	443.09	227.525 to 1865.522							
Acetamiprid	LD_10_	0.47	0.009 to 1.486	1.859	0.363	48.21	2.04	0.326	300	60
	LD_50_	2.30	0.321 to 4.431							
	LD_90_	11.27	6.525 to 24.269							
Copper oxychloride	LD_10_	8.01	2.678 to 13.776	2.260	0.324	156.2	1.50	0.130	298	60
	LD_50_	29.56	18.941 to 39.109							
	LD_90_	109.08	80.277 to 185.305							
Paraffin oil	LD_10_	52.61	44.192 to 58.470	10.556	1.254	79.56	1.41	0.084	297	60
	LD_50_	69.58	63.661 to 74.701							
	LD_90_	92.02	85.209 to 102.718							
Pyriproxyfen	LD_10_	23.24	1.840 to 41.427	1.162	0.413	96.75	0.91	0.485	200	60
	LD_50_	294.42	142.761 to 10,256.807							
	LD_90_	3730.36	681.874 to 41,240,080.332							
Vaseline oil	LD_10_	0.15	0.000 to 1.244	0.786	0.239	114.2	0.68	0.355	200	60
	LD_50_	6.30	0.370 to 14.400							
	LD_90_	269.62	114.416 to 5298.953							
Potassium salt of fatty acids	LD_10_	0.47	0.017 to 1.737	1.102	0.192	127.6	1.53	0.200	250	60
	LD_50_	6.85	1.939 to 12.461							
	LD_90_	99.64	54.482 to 357.491							
Mancozeb	LD_10_	-	-	-	-	-	-	-	300	60
	LD_50_	-	-							
	LD_90_	-	-							

* %RR: Percent of Recommended Rate.

**Table 6:** Test of equality of survival distributions for different temperatures depending on release rate (4 or 8).

	Chi-Square	df	Significance
Log-Rank (Mantel–Cox)	6.687	2	0.035
Breslow (Generalized Wilcoxon)	8.590	2	0.014
Tarone–Ware	8.046	2	0.018

**Table 7:** Mean and median survival times for total predation time under field conditions.

	Mean				Median			
Prey Density	Estimate	Std. Err	95% Confidence Interval		Estimate	Std. Err	95% Confidence Interval	
			Lower Bound	Upper Bound			Lower Bound	Upper Bound
High	132.08	4.63	123.00	141.15	130.00	6.29	117.67	142.33
Low	104.00	9.56	85.26	122.74	111.00	8.38	94.58	127.42
Medium	118.19	4.22	109.92	126.45	118.00	2.43	113.25	122.75

**Table 8:** Pesticides used for compatibility experiment and their recommended rates.

Trade Name	Category	Formulation	Active Ingredient (Amount)	Recommended Rate
AGROIL	Insecticide-Acaricide	Emulsifiable Concentrate (EC)	White oil (78%)	2 L/hL
CITROLE BM	Insecticide-Acaricide	Emulsifiable Concentrate (EC)	Paraffin oil (97%)	1.5 L/hL
SURPOLA	Insecticide	Emulsifiable Concentrate (EC)	Pyriproxyfen (100 g/L)	35 cc/hL
MOSPILAN	Insecticide	Soluble Powder (SP)	Acetamiprid (20%)	20 g/hL
OVIPHYT	Insecticide-Acaricide	Emulsifiable Concentrate (EC)	Vaseline oil (817 g/L)	2 L/hL
BLACK SOAP	Insecticide	-	Potassium salt of fatty acids	2 kg/hL
DITHANE	Fungicide	Wettable Powder (WP)	Mancozeb (80%)	200 g/hL
CUIVROL	Fungicide	Wettable Powder (WP)	Copper oxychloride (50%)	500 g/hL

## Data Availability

All the data are included in this article.

## References

[1] Griffith M.P. (2004). The Origins of an Important Cactus Crop, *Opuntia ficus-indica* (Cactaceae): New Molecular Evidence. Am. J. Bot..

[2] Portillo M.L., Vigueras A.L. (2006). A Review on the Cochineal Species in Mexico, Hosts and Natural Enemies. Acta Hortic..

[3] Bouharroud R., Amarraque A., Qessaoui R. (2016). First Report of the Opuntia Cochineal Scale *Dactylopius opuntiae* (Hemiptera: Dactylopiidae) in Morocco. EPPO Bull..

[4] Arias-Estévez M., López-Periago E., Martínez-Carballo E., Simal-Gándara J., Mejuto J.C., García-Río L. (2008). The Mobility and Degradation of Pesticides in Soils and the Pollution of Groundwater Resources. Agric. Ecosyst. Environ..

[5] Badii M.H., Flores A.E. (2001). Prickly Pear Cacti Pests and Their Control in Mexico. Fla. Entomol..

[6] Bouharroud R., Hanafi A., Serghini M.A. (2007). Pyrethroids and Endosulfan Resistance of *Bemisia tabaci* in the Tomato Greenhouses of the Souss Valley of Morocco. Acta Hortic..

[7] Akroud H., Sbaghi M., Bouharroud R., Koussa T., Boujghagh M., El Bouhssini M. (2021). Antibioisis and Antixenosis Resistance to *Dactylopius opuntiae* (Hemiptera: Dactylopiidae) in Moroccan Cactus Germplasm. Phytoparasitica.

[8] El Aalaoui M., Bouharroud R., Sbaghi M., El Bouhssini M., Hilali L. (2019). Predatory Potential of Eleven Native Moroccan Adult Ladybird Species on Different Stages of *Dactylopius opuntiae* (Cockerell) (Hemiptera: Dactylopiidae). EPPO Bull..

[9] Vanegas-Rico J.M., Rodríguez-Leyva E., Lomeli-Flores J.R., González-Hernández H., Pérez-Panduro A., Mora-Aguilera G. (2016). Biology and Life History of *Hyperaspis trifurcata* Feeding on *Dactylopius opuntiae*. BioControl.

[10] Bouharroud R., Sbaghi M., Boujghagh M., El Bouhssini M. (2018). Biological Control of the Prickly Pear Cochineal *Dactylopius opuntiae* Cockerell (Hemiptera: Dactylopiidae). EPPO Bull..

[11] Hernández-González I.A., Cruz-Rodríguez J.A. (2018). *Chilocorus cacti* (Coleoptera: Coccinellidae) as a Biological Control Agent of the Wild Cochineal (Hemiptera: Dactylopiidae) of Prickly Pear Cactus. Environ. Entomol..

[12] Vanegas-Rico J.M., Lomeli-Flores J.R., Rodríguez-Leyva E., Pérez-Panduro A., González-Hernández H., Marín-Jarillo A. (2015). *Hyperaspis trifurcata* (Coleoptera: Coccinellidae) and Its Parasitoids in Central Mexico. Rev. Colomb. Entomol..

[13] Vanegas-Rico J.M., Lomelí-Flores J.R., Rodríguez-Leyva E., Mora-Aguilera G., Valdez J.M. (2010). Enemigos Naturales de *Dactylopius opuntiae* (Cockerell) En Opuntia Ficus-Indica (L.) Miller En El Centro de México. Acta Zool. Mex..

[14] Bouharroud R., El Aalaoui M., Boujghagh M., Hilali L., El Bouhssini M., Sbaghi M. (2019). New Record and Predatory Activity of *Hyperaspis campestris* (Herbst 1783) (Coleoptera: Coccinellidae) on *Dactylopius opuntiae* (Hemiptera: Dactylopiidae) in Morocco. Entomol. News.

[15] Cruz-Rodríguez J.A., González-Machorro E., Villegas González A.A., Rodríguez Ramírez M.L., Mejía Lara F. (2016). Autonomous Biological Control of *Dactylopius opuntiae* (Hemiptera: Dactyliiopidae) in a Prickly Pear Plantation with Ecological Management. Environ. Entomol..

[16] Gilreath M.E., Smith J.W. (1988). Natural Enemies of *Dactylopius confusus* (Homoptera: Dactylopiidae): Exclusion and Subsequent Impact on Opuntia (Cactaceae). Environ. Entomol..

[17] Vanegas-Rico J.M., Pérez-Panduro A., Lomelí-Flores J.R., Rodríguez-Leyva E., Valdez-Carrasco J.M., Mora-Aguilera G. (2017). *Dactylopius opuntiae* (Cockerell) (Hemiptera: Dactylopiidae) population fluctuations and predators in Tlalnepantla, Morelos, Mexico. Folia Entomol. Mex. (Nueva Ser.).

[18] Ascencio-Contreras D.O., Alvarado-Gómez O.G., Lara-Ávila J.P., Jarquín-Gálvez R., Ávila-Rodríguez V. (2020). Aspectos Biológicos de Coccinélidos Asociados a *Opuntia ficus indica* En San Luis Potosí, México. Southwest. Entomol..

[19] Barreto-García O.A., Rodríguez-Leyva E., Lomeli-Flores J.R., Vanegas-Rico J.M., Vigueras A.L., Portillo L. (2020). *Laetilia coccidivora* Feeding on Two Cochineal Insect Species, Does the Prey Affect the Fitness of the Predator?. BioControl.

[20] Aldama-Aguilera C., Llanderal-Cázares C., Soto-Hernández M., Castillo-Márquez L.E. (2005). Producción de grana-cochinilla (Dactylopius Coccus Costa) en plantas de nopal a la intemperie y en microtúneles. Agrociencia.

[21] Ascencio Contreras D.O. (2021). Coccinélidos Como Enemigos Naturales de *Dactylopius opuntiae* (Cockerell). Ph.D. Thesis.

[22] Eisner T., Ziegler R., McCormick J.L., Eisner M., Hoebeke E.R., Meinwald J. (1994). Defensive Use of an Acquired Substance (Carminic Acid) by Predaceous Insect Larvae. Experientia.

[23] Cottrell T.E. (2005). Predation and Cannibalism of Lady Beetle Eggs by Adult Lady Beetles. Biol. Control..

[24] Hemptinne J.L., Lognay G., Gauthier C., Dixon A.F.G. (2000). Role of Surface Chemical Signals in Egg Cannibalism and Intraguild Predation in Ladybirds (Coleoptera: Coccinellidae). Chemoecology.

[25] Snyder W.E., Joseph S.B., Preziosi R.F., Moore A.J. (2000). Nutritional Benefits of Cannibalism for the Lady Beetle *Harmonia axyridis* (Coleoptera: Coccinellidae) When Prey Quality Is Poor. Environ. Entomol..

[26] Ponsonby D.J., Copland M.J.W. (2007). Influence of Host Density and Population Structure on Egg Production in the Coccidophagous Ladybird, *Chilocorus nigritus* F. (Coleoptera: Coccinellidae). Agric. For. Entomol..

[27] Mena Covarrubias J., Rosas Gallegos S. (2007). Guía Para El Manejo Integrado de Las Plagas Del Nopal Tunero.

[28] Bogdanova N.L. (1956). *Hyperaspis campestris* Herbst (Coleoptera: Coccinellidae) as Destroyer of *Chloropulvinaria floccifera* Westwood.(Homoptera: Coccidoidea). Entomol. Obozr..

[29] Mendel Z., Protasov A., Vanegas-Rico J.M., Lomeli-Flores J.R., Suma P., Rodríguez-Leyva E. (2020). Classical and Fortuitous Biological Control of the Prickly Pear Cochineal, *Dactylopius opuntiae*, in Israel. Biol. Control..

[30] Reyd G., Le Rü B. (1992). Influence de La Prédation Des Larves d’*Hyperaspis Raynevali* et d’*Hexochomus flaviventris* (Col. Coccinellidae) Sur Les Colonies de La Cochenille Du Manioc *Phenacoccus manihoti* (Hom. Pseudococcidae). Étude En Conditions Contrôlées. Entomophaga.

[31] Seyfollahi F., Esfandiari M., Mossadegh M.S., Rasekh A. (2016). Life Table Parameters of the Coccinellid *Hyperaspis polita*, a Native Predator in Iran, Feeding on the Invasive Mealybug *Phenacoccus Solenopsis*. J. Asia Pac. Entomol..

[32] Trouve C., Ledee S., Ferran A., Brun J. (1997). Biological Control of the Damson-Hop Aphid, *Phorodon humuli* (Hom.: Aphididae), Using the Ladybeetle *Harmonia axyridis* (Col.: Coccinellidae). Entomophaga.

[33] Crowder D.W. (2007). Impact of Release Rates on the Effectiveness of Augmentative Biological Control Agents. J. Insect Sci..

[34] Juliano S.A., Scheiner S.M., Gurevitch J. (2001). Non-linear curve fitting: Predation and functional response curves. Design and Analysis of Ecological Experiments.

[35] Zhang L., Lv H., Li X., Wan H., He S., Li J., Ma K. (2023). Sublethal Effects of Acetamiprid and Afidopyropen on Harmonia Axyridis: Insights from Transcriptomics Analysis. Ecotoxicol. Environ. Saf..

[36] Najar-Rodríguez A.J., Lavidis N.A., Mensah R.K., Choy P.T., Walter G.H. (2008). The Toxicological Effects of Petroleum Spray Oils on Insects–Evidence for an Alternative Mode of Action and Possible New Control Options. Food Chem. Toxicol..

[37] Shannon B., Walker E., Johnson R.M. (2023). Toxicity of Spray Adjuvants and Tank Mix Combinations Used in Almond Orchards to Adult Honey Bees (*Apis mellifera*). J. Econ. Entomol..

